# Reducing Off-State and Leakage Currents by Dielectric Permittivity-Graded Stacked Gate Oxides on Trigate FinFETs: A TCAD Study

**DOI:** 10.3390/mi15060726

**Published:** 2024-05-30

**Authors:** Alper Ülkü, Esin Uçar, Ramis Berkay Serin, Rifat Kaçar, Murat Artuç, Ebru Menşur, Ahmet Yavuz Oral

**Affiliations:** 1 Department of Material Science and Engineering, Gebze Technical University, Kocaeli 41400, Türkiye; eucar@aselsan.com.tr (E.U.); rbserin@aselsan.com.tr (R.B.S.); ebrualkoy@gtu.edu.tr (E.M.); aoral@gtu.edu.tr (A.Y.O.); 2 Microelectronics, Guidance and Electro-Optics Business Sector, ASELSAN, Akyurt, Ankara 06750, Türkiye; rifatkacar@aselsan.com.tr (R.K.); muratartuc@aselsan.com.tr (M.A.)

**Keywords:** graded dielectric permittivity gate oxides, κ-graded stacked gate oxides, dielectric permittivity matching, grading profile, effective dielectric constant (κ_EFF_), Penn Model, Maxwell–Garnett mixing formula, SILVACO ATLAS, Fin-Field Effect Transistors (FinFET)

## Abstract

Since its invention in the 1960s, one of the most significant evolutions of metal-oxide semiconductor field effect transistors (MOSFETs) would be the 3D version that makes the semiconducting channel vertically wrapped by conformal gate electrodes, also recognized as FinFET. During recent decades, the width of fin (W_fin_) and the neighboring gate oxide width (t_ox_) in FinFETs has shrunk from about 150 nm to a few nanometers. However, both widths seem to have been leveling off in recent years, owing to the limitation of lithography precision. Here, we show that by adapting the Penn model and Maxwell–Garnett mixing formula for a dielectric constant (κ) calculation for nanolaminate structures, FinFETs with two- and three-stage κ-graded stacked combinations of gate dielectrics with SiO_2_, Si_3_N_4_, Al_2_O_3_, HfO_2_, La_2_O_3_, and TiO_2_ perform better against the same structures with their single-layer dielectrics counterparts. Based on this, FinFETs simulated with κ-graded gate oxides achieved an off-state drain current (I_OFF_) reduced down to 6.45 × 10^−15^ A for the Al_2_O_3_: TiO_2_ combination and a gate leakage current (I_G_) reaching down to 2.04 × 10^−11^ A for the Al_2_O_3_: HfO_2_: La_2_O_3_ combination. While our findings push the individual dielectric laminates to the sub 1 nm limit, the effects of dielectric permittivity matching and κ-grading for gate oxides remain to have the potential to shed light on the next generation of nanoelectronics for higher integration and lower power consumption opportunities.

## 1. Introduction

Silicon oxide has been used as a gate dielectric material on thin film transistors for over 40 years, but as dimensions shrink, alternatives with higher dielectric constants are necessary to reduce leakage currents. While high-κ dielectrics have been investigated for their thermal stability and compatibility with Si, FinFET technology, with 3D double-gate and triple-gate transistors, has further advanced, leading to smaller, more efficient transistors with reduced power consumption [1,2,3,4,5].

The continuous downscaling of MOS devices is indispensable for increasing the transistor density and performance, leading to efficient chip functionality at higher speeds. However, this scaling poses challenges such as severe short channel effects (SCEs), increased fabrication costs, and difficulties in device processing [6,7,8]. Multi-gate MOS device structures like FinFETs, which use multiple gate electrodes and an ultrathin body, have been developed to address these challenges, showing an excellent device performance at scaled parameters. The use of metal gates has become attractive due to their chemical stability with high-κ gate dielectrics and the ability to maintain higher threshold voltages while acquiring high gate stack stability [9,10,11,12,13,14].

Research on stacked gate dielectrics on thin film transistors first appeared in 1994 by Kuo when SiN_x_ laminates with different dielectric deposition conditions were experimented and compared with single SiN_x_ as the gate dielectric [15]. This paper analyzed how TFT mobility, V_TH_, SS, I_ON,_ and I_OFF_ was affected due to different gas flow concentrations in the PECVD process to develop the SiN_x_ layer. Regarding gate dielectrics consisting of two- or three-stage known dielectrics working on FinFETs, fabrications on top of Si-channel FinFETs were presented in papers by Dosev [16] in 2003 and by Jankovic [17] in 2012. Kauerauf [18] in 2005 tried to minimize the gate leakage current by using SiO_2_ and various high-κ dielectrics like ZrO_2_ and HfO_2_ together in the same stack. In 2019, Das et al. [19] proposed a dual-material-gate, dual-stacked-gate dielectrics and gate-source-overlapped Germanium FinFET with a low leakage I_G_ current, high I_D_ current, and high drain current ratio I_ON_/I_OFF_. Gangwani et al. [20] analyzed the temperature performance of a stacked SiO_2_: HfO_2_-gated FinFET, which showed an enhanced output performance and reduced short channel effects compared to the conventional FinFET in 2022.

In a patent by Gardner [21] in 2000, a three-layer graded dielectric film was formed on an upper surface of the semiconductor substrate. A second dielectric film of SiN_x_ was deposited on the first dielectric film and a third dielectric film of oxide of one of the elements Be, Mg, Ca, Ti, Zr, or Ta was then deposited on the surface of the second dielectric film. All dielectric films were then annealed along with the semiconductor substrate by immersing into an inert ambient maintained at a temperature in the range of approximately 600–1100 °C. This work was the main cornerstone and first sign of commercialization of the graded dielectric research upon thin film transistors and was followed by a patent by Kang [22] applied by Samsung in 2011 on the employing of a graded metal oxide layer for planar transistors and another patent by Gealy [23] applied by Micron Technology on graded dielectric structures in 2017.

Simulation wise, on heterogated structures, SILVACO ATLAS and many other simulation tools are employed with many standard recombination and continuity models like Shockley–Read–Hall, Schrödinger, and Auger, which are used widespread for 2D/3D simulations of normal or hetero-gated single-, double-, or triple-gated FinFETs in [24,25,26]. Bousari [27] demonstrated, in simulations with this tool, that hetero-gated dielectric structures of SiO_2_, Si_3_N_4_, Al_2_O_3_, and HfO_2_ enable a significant performance increase on dual- and triple-gate FinFETs. Vijaya [28], again via the same tool, exercised single-layer SiO_2_, Si_3_N_4_, HfO_2_, and TiO_2_ gate oxides upon 32 nm silicon-on-insulator (SOI) FinFET, where HfO_2_ and TiO_2_ usage significantly enhanced the device I_ON_ and transconductance. Saha [29], in 2023, performed the optimization and analysis of a triple-fin Heterostructure-on-Insulator (HOI) with a dual-stacked gate oxide combination using SiO_2_, Si_3_N_4_, Al_2_O_3_, HfO_2_, and ZrO_2_ dielectrics at a 10 nm FinFET. Vimala [30] performed simulations using gate metal engineering with Co, W, and Al together on a trigate FinFET. Nagy et al. [31] explored nanowire FET architectures through a simulation in a VENDES finite element toolbox that integrated Schrödinger equation-based quantum corrected methods. Garduño [32] modeled gate leakage currents for many FinFET structures and the implementation was performed in Verilog-A.

Even though these studies demonstrated multi-material stacked gate oxides’ potential to function as better gate insulators, the process of the selection of the material and the related thickness engineering have appeared rather ad hoc, arbitrary, or merely by past research experience, which overlooked measuring or to calculating the resultant dielectric permittivity, $\kappa_{EFF}$, of the stacked gate oxide structure.

According to Giustino, Peng, and Wang [33,34,35,36], dielectric permittivity matching reduces strain especially at insulator interfaces aiding in minimizing interface stress. Even if metals hypothetically have bulk dielectric permittivity of near infinity, they tend to have dielectric permittivity values closer to ceramics and oxides when their thicknesses are limited to a few nanometers [37,38,39,40,41].

The permittivity matching TFT designs appeared [42,43,44] when the SiO_2_ and SiN_x_ gate insulators were discovered to be behaving well when neighboring the Si channel [44], and designers frequently used the Equivalent Oxide Thickness (EOT) convention [41,45,46,47] for the determination of the thickness of hi-κ gate oxide to replace the SiO_2_ or SiN_x_. But EOT also had its disadvantages, like its invalidity for non-planar devices due to the impact of device geometry on capacitance behavior [48] and a gate-leakage current increase when the gate oxide layer is scaled down below 2 nm [49].

With κ-grading (also called as “epsilon grading” (ε-grading), so that dielectric permittivity changes through device depth is interchangeably designated as “ε” or “κ” in different references), our aim is to match the dielectric permittivity of stages; i.e., the Si channel is followed by a dielectric material with the lowest bulk dielectric constant $\kappa_{b}$, followed by a material with a higher $\kappa_{b}$, then followed by a material with a higher $\kappa_{b}$ again, until the gate is reached. κ-grading together with an effective dielectric constant ($\kappa_{EFF}$) calculation of the staged/graded gate oxide structure is proposed for the better effectivity of gate oxide. We highlight three steps in the incorporation of this technique as follows:

1. κ-grading is employed for stacked gate oxide. This is detailed in Section 3.1.1.
2. Even when a single material gate dielectric is used, the Penn model [50,51] can be utilized for the calculation of effective dielectric constants of the gate oxide layer, $\kappa_{EFF}$, as the bulk dielectric constant usage will be misleading for gate oxides with thicknesses of a few nanometers. This is detailed in Section 3.1.2.
3. With each addition of a new laminate material, the overall effective dielectric constant of the gate oxide layer, $\kappa_{EFF}$, can be recalculated using the Maxwell–Garnett [52] mixing formula, so that a fair mechanism is established to compare the performance of FinFETs with respect to this $\kappa_{EFF}$ as the independent variable. The mentioned calculations are given in Section 3.1.3.

Our research work offers the most comprehensive simulation work in the investigation of stacked gate oxides on FinFETs with 41 different gate oxide combinations, all with a 3 nm total thickness, adding two-stage or three-stage κ-grading features and taking an effective dielectric constant ($\kappa_{EFF}$) calculation into account. In this paper, we present the simulation results obtained using SILVACO ATLAS for a 3D silicon on insulator (SOI) n-FinFET structure with κ-graded stacked gate oxides.

This manuscript is divided into several sections: In Section 2, the FinFET device structure, its geometry and gate dielectric combinations, and their designations are introduced. In Section 3, details of the κ-grading, effective dielectric constant $\kappa_{EFF}\;$ calculation, mathematical methods for FinFET modeling, simulation tool usage, and choice of performance metrics are presented. Our simulation results are exhibited and discussed with some analysis and insights that we derived in Section 4, Section 5 and Section 6. Finally, fabrication considerations and the conclusions are reported in Section 7 and Section 8.

## 2. Device Structure

### 2.1. FinFET Geometric Model

**Figure 1:**
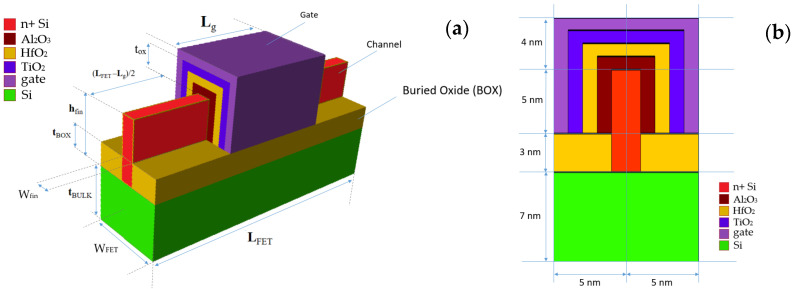
(**a**) gκ-FinFET geometric model with 3-stage κ-graded gate oxide with thickness t_ox_, (**b**) inset of the cross-section, with geometry parameters in Table 1.

The 3D Technology Computer-Aided Design (TCAD) structure for a FinFET with a gate oxide with graded dielectric permittivity is shown in Figure 1. Using SILVACO ATLAS for device simulation and with a gate oxide thickness (t_ox_) of 3 nm, the buried oxide (BOX) material is kept as HfO_2_ and never changed through all simulations. An equal doping concentration (N_d_) of 5 × 10^19^ cm^−3^ is the used source–drain channel region. Other FinFET properties are shown in Table 1. We call this FinFET type “FinFET with κ-graded gate oxide” or “gκ-FinFET” throughout the paper. The device structure is of an n-type FinFET, comprising three gates, one on top and two at the sides of the fin-shaped channel, not isolated, but behaving as a single inversed U-shaped gate. Metal with a work function (*ϕ_w_*) of 5 eV is applied at the gate, common for n+-doped Si channel junctionless architectures [7,27,28]. Ni or CrAu alloy is suitable for this work function value, common for junctionless n-TFTs.

### 2.2. Gate Dielectrics

Six base dielectric materials, SiO_2_, Si_3_N_4_, Al_2_O_3_, HfO_2_, La_2_O_3_, and TiO_2_, bulk dielectric constants of which are shown in Table 2, are selected as single-layer gate dielectrics of a 3 nm thickness (t_ox_) for a 14 nm channel length (L_FET_) gκ-FinFET structure. These six materials are used one-by-one for first six simulations to form the control group.

Then 15 different two-stage and 20 different three-stage κ-graded material combinations composed of these six base dielectrics, as designated in Table 3, are devised between the Si channel and the gate. The **AHT** case consists of Al_2_O_3_: HfO_2_: TiO_2_ gate oxides, as shown in Figure 1.

**Table 2:** Bulk dielectric constant of gate oxide materials [53].

Dielectric Material	$\kappa_{b}$
SiO_2_	3.9
Si_3_N_4_	7.4
Al_2_O_3_	9
HfO_2_	25
La_2_O_3_	30
TiO_2_	95

**Table 3:** gκ-FinFET reference designators for single and compound gate oxides of 41 simulations.

Gate Oxide Type	Dielectric Material Combination	FinFET Reference Designator
Single-material gate oxide	SiO_2_	**S1**
	Si_3_N_4_	**S2**
	Al_2_O_3_	**A**
	HfO_2_	**H**
	La_2_O_3_	**L**
	TiO_2_	**T**
Dual-material κ-graded gate oxide	SiO_2_: Si_3_N_4_	**S1S2**
	SiO_2_: Al_2_O_3_	**S1A**
	SiO_2_: HfO_2_	**S1H**
	SiO_2_: La_2_O_3_	**S1L**
	SiO_2_: TiO_2_	**S1T**
	Si_3_N_4_: Al_2_O_3_	**S2A**
	Si_3_N_4_: HfO_2_	**S2H**
	Si_3_N_4_: La_2_O_3_	**S2L**
	Si_3_N_4_: TiO_2_	**S2T**
	Al_2_O_3_: HfO_2_	**AH**
	Al_2_O_3_: La_2_O_3_	**AL**
	Al_2_O_3:_ TiO_2_	**AT**
	HfO_2:_ La_2_O_3_	**HL**
	HfO_2_: TiO_2_	**HT**
	La_2_O_3_: TiO_2_	**LT**
Triple-material κ-graded gate oxide	SiO_2_: Si_3_N_4_: Al_2_O_3_	**S1S2A**
	SiO_2_: Si_3_N_4_: HfO_2_	**S1S2H**
	SiO_2_: Si_3_N_4_: La_2_O_3_	**S1S2L**
	SiO_2_: Si_3_N_4_: TiO_2_	**S1S2T**
	SiO_2_: Al_2_O_3_: HfO_2_	**S1AH**
	SiO_2_: Al_2_O_3_: La_2_O_3_	**S1AL**
	SiO_2_: Al_2_O_3_: TiO_2_	**S1AT**
	SiO_2_: HfO_2_: La_2_O_3_	**S1HL**
	SiO_2_: HfO_2_: TiO_2_	**S1HT**
	SiO_2_: La_2_O_3_: TiO_2_	**S1LT**
	Si_3_N_4_: Al_2_O_3_: HfO_2_	**S2AH**
	Si_3_N_4_: Al_2_O_3_: La_2_O_3_	**S2AL**
	Si_3_N_4_: Al_2_O_3_: TiO_2_	**S2AT**
	Si_3_N_4_: HfO_2_: La_2_O_3_	**S2HL**
	Si_3_N_4_: HfO_2_: TiO_2_	**S2HT**
	Si_3_N_4_: La_2_O_3_: TiO_2_	**S2LT**
	Al_2_O_3_: HfO_2_: La_2_O_3_	**AHL**
	Al_2_O_3_: HfO_2_: TiO_2_	**AHT**
	Al_2_O_3_: La_2_O_3_: TiO_2_	**ALT**
	HfO_2_: La_2_O_3_: TiO_2_	**HLT**

In Table 3, we introduce reference designators in the last column for gκ-FinFET equipped with each gate oxide material for the easy reading of the figures incorporated in the results. The designator consists of two to four alphanumeric characters, including the first character of each gate oxide it consists of. Since SiO_2_ and Si_3_N_4_ have the same first character, gκ-FinFETs with their respective gate oxides were designated as **S1** and **S2**, respectively. All the parameters for gκ-FinFET were kept the same at each simulation, only the gate oxide layer material combination was changed, making a total of 41 simulations. The performances of the FinFETs with these gate oxide combinations, will be shown in subsequent pages and can be followed with these designations which appear in **boldface** throughout the paper and the individual stage thicknesses read from Table 4. For example, FinFET with a gate oxide of a single layer of SiO_2_ is designated as **S1**, the same with a single layer of Si_3_N_4_ as **S2**; for the Al_2_O_3_: TiO_2_ gate oxide combination, the FinFET is designated as **AT**, and for a Si_3_N_4_: La_2_O_3_: TiO_2_ combination, the same is designated as **S2LT**.

## 3. Methods

Our methods, mathematical derivations and modeling, choice of performance metrics, and usage of these figures of merit (FoM) for evaluation are presented herein with following main steps:

○ κ-grading and calculation of effective κ of the gate oxide.
○ Mathematical modeling in ATLAS Software v5.34.0.R.
○ Choice of performance metrics for performance evaluation.

### 3.1. κ-Grading and Calculation of Effective κ for Gate Oxides

#### 3.1.1. κ-Grading

**Figure 2:**
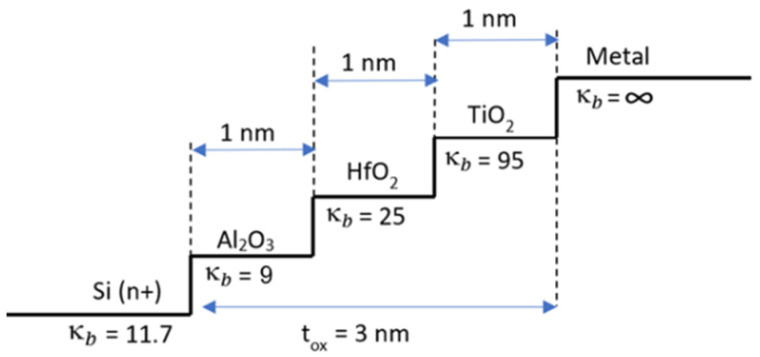
Stepwise κ-graded stacked gate oxide profile of **AHT** gκ-FinFET.

Regarding κ-grading, we mean that, among selected dielectric materials to be used for stacking, Si channel deposition should be followed by dielectric material with lowest bulk dielectric constant $\kappa_{b}$, followed by material with higher $\kappa_{b}$, then followed by a material with higher $\kappa_{b}$ again, until gate is reached like in Figure 2. We mainly target dielectric permittivity matching of gate oxide at both ends of Si channel side and metal side. Thus, as permittivity matching at both ends of the gate oxide is considered, we implement this concept herein by κ-grading, keeping permittivity of neighboring materials as close as possible.

#### 3.1.2. Penn Model: Calculation of κ for Each Nanolaminate

**Figure 3:**
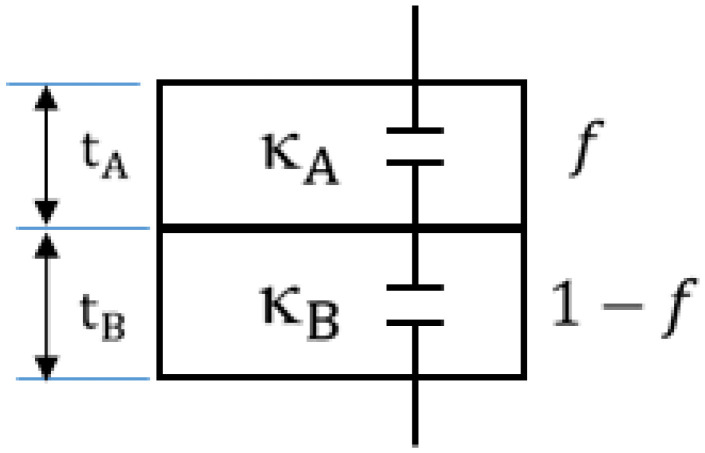
Two-phase dielectric system connected in series in parallel sheets.

Suppose $\kappa_{bA},\;\kappa_{bB}$ are bulk dielectric constants for materials A and B and $\kappa_{A},\;\kappa_{B}$ are calculated dielectric constants of their respective nanolaminates with f*,* the volumetric filling factor for material A, and 1−f is the volumetric filling factor for material B, in a two-phase dielectric system of Figure 3.

A theoretical foundation was first given by Penn’s 1962 paper [50]. For Si, it has been shown that for thicknesses greater than 200 Å (20 nm), bulk $\kappa_{bA}$ can be considered to be unchanged and equivalent to $\kappa_{A}$, and if $t_{A}$ is less than 200 Å, one needs to consider using the wave number dependence equation for changing dielectric function. For practical purposes, this equation evolved into a modified model [54] by Tsu in 1997, and then into a generalized one [51] by Sharma in 2006, for calculation of size-dependent energy gap and dielectric permittivity of nanolaminated dielectric structures under quantum confinement effects, where $\kappa_{A}$ becomes less than $\kappa_{bA}$. A patent by Gealy [23] in 2012 incorporated similar equations to calculate the dielectric constant of thin nanolaminate, as stated in Equation (1). Our FinFET under consideration requires 1 nm, 1.5 nm, and 3 nm gate oxide nanolaminates; we chose to use Sharma’s generalized Penn model. Calculation of effective κ, hereinafter $\kappa_{EFF},\;$ of this dielectric system in case of any narrowed individual thickness $t_{A}$ or $t_{B}$ below 200 Å is presented in two steps:

First, nanolaminate dielectric constant $\kappa_{A}$ due to thickness $t_{A}$ of nanometer order is to be calculated by Equation (1):

(1) $$\kappa_{A}=1+\frac{\kappa_{bA}-1}{1+(\frac{\kappa_{\infty A}}{K_{fA}t_{A}})}$$

where $\kappa_{bA}$ is the bulk dielectric constant, $\kappa_{\infty A}$ is the high-frequency dielectric constant, $K_{fA}$ is Fermi wave vector, and $t_{A}$ is the planar thickness of the nano-scaled dielectric material A. Equation (1) can be numerically generalized and further fitted to Equation (2) as in [51], forming the generalized Penn Model which we utilize for our calculations of $\kappa_{A}$ for desired thickness $t_{A}$:

(2) $$\kappa_{A}=1+\frac{\kappa_{bA}-1}{1+1.7{t_{A}}^{-1.8}}$$

**Figure 4:**
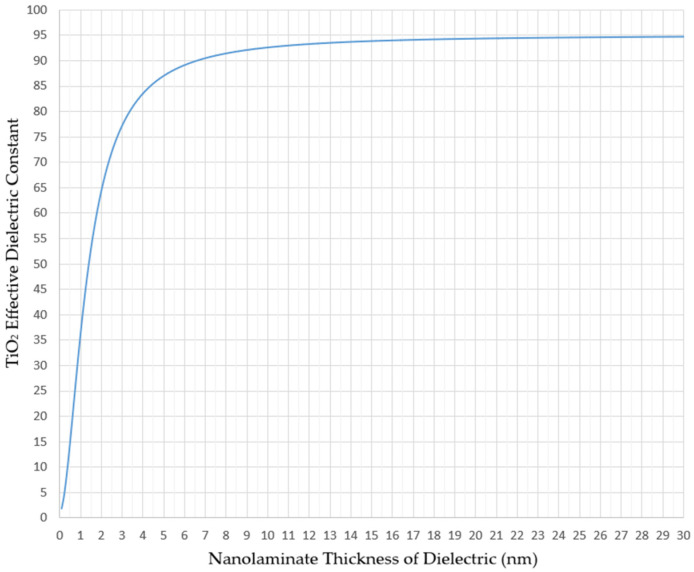
$\kappa_{A}$ with respect to TiO_2_ thickness calculated by Equation (2).

When we calculate the resultant $\kappa_{A}$ of material due to its nanolaminate thickness $t_{A}$, we observe significant loss in dielectric effect. This numerical approximation is depicted in Figure 4 for TiO_2_ material, showing that in orders of few nanometers, $\kappa_{A}$ reduction is significant. At 3 nm thickness, $\kappa_{A}$ becomes 77, at 1.5 nm it is 52.6, and at 1 nm it is 35.8 when compared to its bulk value of 95.

#### 3.1.3. Maxwell–Garnett Model: Calculation of κ for Whole Gate Oxide

Dielectric constant $\kappa_{EFF}$ of system of nanolaminates due to thickness $t_{ox}=t_{A}+t_{B}$ and with volumetric filling factor is calculated by Maxwell–Garnett mixing formula.

(3) $$\kappa_{EFF,\;\;AB}=\kappa_{B}\frac{\kappa_{A}+2\kappa_{B}+2f_{A}(\kappa_{A}-\kappa_{B})}{\kappa_{A}+2\kappa_{B}-2f_{A}(\kappa_{A}-\kappa_{B})}$$

Niklasson et al. [55] used, in 1981, the Maxwell–Garnett and Bruggeman effective medium theories to derive average dielectric permeability of heterogeneous materials and estimated dielectric properties of a composite material composed of Cobalt and Alumina. Petrovsky [56] laid foundations of multi-material “effective dielectric constant” calculation with profound detail in 2012 mainly by Bruggeman equations with respect to volumetric filling factor f. Markel [52] in 2016 issued a framework tutorial, surveying existing methods and restating the Maxwell–Garnett mixing formula for calculation of $\kappa_{EFF}$ for two-stage dielectrics. This formula gives the effective permittivity in terms of the permittivity and volume fractions of the individual constituents of the complex medium and is shown in Equation (3).

To extend this formula for a three-phase system, we denote the dielectric constants of the three materials as $\kappa_{A}$, $\kappa_{B}$, and $\kappa_{C}$ and their respective volumetric filling factors as $f_{A}$, $f_{B}$, and $f_{C}$ where $f_{A}$ + $f_{B}$ + $f_{C}$ = 1, and we need to simply derive the same equation that considers all three materials. Thus, we can now:

- Calculate the effective dielectric constant $\kappa_{AB}$ for materials A and B using the Maxwell–Garnett mixing formula.
- Consider $\kappa_{AB}$ as single-material AB’s dielectric constant and apply the Maxwell–Garnett formula again, with input variables $\kappa_{AB}$ and $\kappa_{C}$, to find the overall effective dielectric constant $\kappa_{EFF}$, with $f_{AB}$ + $f_{C}$ = 1, where $f_{AB}$ = $f_{A}$ + $f_{B}$, and finally, our equation becomes Equation (4) for a complex medium of three phases, A, B, and C.
  (4) $$\kappa_{EFF,\;\;AB}=\kappa_{B}\frac{\kappa_{AB}+2\kappa_{C}+2f_{AB}(\kappa_{AB}-\kappa_{C})}{\kappa_{AB}+2\kappa_{C}-2f_{AB}(\kappa_{AB}-\kappa_{C})}$$

Therefore, using Equations (3) and (4), we calculated the $\kappa_{EFF}$ of two-stage and three-stage dielectric materials denoted in last column of Table 4.

### 3.2. Mathematical Models in ATLAS

This section lays out modeling methods we utilize in ATLAS, Non-Equilibrium Green’s Function, Hot Electron/Hole Injection Model and Direct Quantum Tunneling Model, equations of which are employed within simulations.

#### 3.2.1. Quantum Transport: Non-Equilibrium Green’s Function (NEGF) Approach

This fully quantum method treats such effects as source-to-drain tunneling, ballistic transport, and quantum confinement on equal footing. This situation is common to double gate and trigate transistors, FinFETs, and nanowire FETs.

By specifying the NEGF_MS and SCHRODINGER options on the MODELS statement, we can launch a NEGF solver to model ballistic quantum transport in such devices as double gate or surround gate MOSFET. An effective-mass Hamiltonian $H_{o}\;$ of a two-dimensional device is given by:

(5) $$H_{o}=-\frac{h^{2}}{2}\left[\frac{\partial }{\partial x}\left(\frac{1}{m_{x}^{v}(x,y)}\frac{\partial }{\partial x}\right)+\frac{\partial }{\partial y}\left(\frac{1}{m_{y}^{v}(x,y)}\frac{\partial }{\partial y}\right)\right]$$

when discretized in real space using a finite volume method. A corresponding expression in cylindrical coordinates is:

(6) $$H_{o}=-\frac{h^{2}}{2}\left[\frac{1}{r}\frac{\partial }{\partial r}\left(\frac{1}{m_{r}^{v}(r,z)}r\frac{\partial }{\partial r}\right)-\frac{1}{m_{r}^{v}\left(r,z\right)}\frac{m^{2}}{r^{2}}+\frac{\partial }{\partial z}\left(\frac{1}{m_{z}^{v}(r,z)}\frac{\partial }{\partial z}\right)\right]$$

Rather than solving a 2D or 3D problem, which may take vast amounts of computational time, a Mode Space (MS) approach is used. A Schrodinger equation is first solved in each slice of the device to find eigenenergies and eigenfunctions. Then, a transport equation of electrons moving in the sub-bands is solved. As only a few lowest eigen sub-bands are occupied and the upper sub-bands can be safely neglected, the size of the problem is reduced. In the devices where the cross-section does not change, the sub-bands are not quantum-mechanically coupled to each other, and the transport equations become essentially 1D for each sub-band. Therefore, we can further divide the method into Coupled (CMS) or Uncoupled Mode Space (UMS) approaches. ATLAS tool automatically decides on the minimum number of sub-bands required and the method to be used. It is possible, however, to set the number of sub-bands by using the EIGEN parameter on the MODELS statement. To enforce either CMS or UMS approaches, we can use NEGF_CMS or NEGF_UMS instead of NEGF_MS on the MODELS statement. The transformation of a real space Hamiltonian $H_{o}\;$ to a mode space is done by taking a matrix element between *m^th^* and *n^th^* wave functions of *k^th^* and *l^th^* slices:

(7) $$H_{mnkl}^{MS}=\left\langle \Psi_{m}^{k}(y)|H_{o}|\Psi_{n}^{l}(y)\right\rangle$$

Skipping some middle steps of derivation from [57], 2-dimensional carrier density and corresponding current density functions are laid as follows:

Carrier density function:

(8) $$n\left(x_{i},y_{i}\right)=-\frac{i}{hL_{z}}\sum_{k_{2}\sigma }\sum_{mn}\int G_{mnii}^{<}\left(E\right)\Psi_{m}^{i}(y_{j})\Psi_{n}^{*i}(y_{j})\frac{dE}{2\pi }$$

x-component of current density:

(9) $$J_{x}\left(x_{i},y_{i}\right)=-\frac{2e}{hL_{z}{∆}_{x}}\sum_{k_{2}\sigma }\sum_{mn}\int Re(t_{i+1jjj}G_{mnii+1}^{<}\left(E\right))\Psi_{m}^{i}(y_{j})\Psi_{n}^{*i+1}(y_{j})\frac{dE}{2\pi }$$

y-component of current density:

(10) $$J_{y}\left(x_{i},y_{i}\right)=-\frac{2e}{hL_{z}{∆}_{y}}\sum_{k_{2}\sigma }\sum_{mn}\int Re(t_{iijj+1}+G_{mnii}^{<}\left(E\right))\Psi_{m}^{i}(y_{j})\Psi_{n}^{*i}(y_{j+1})\frac{dE}{2\pi }$$

Total current density:

(11) J=Jx2+Jy212

Here, *G^<^* is the Green’s function as a matrix, whose diagonal elements are carrier densities as function of energy. *t_ijkl_* is an off-diagonal element of real space Hamiltonian $H_{o}$, which couples nodes (*x_i_,y_k_*) and (*x_j_,y_l_*). In our overall model, this current density *J* is to be integrated through the model geometry to yield the total current that will add up with the currents calculated by other models stated in next two sections.

#### 3.2.2. Lucky-Electron Hot Carrier Injection Model

The Lucky-Electron Model (LEM), proposed in 1984 by Tam, Ko, and Hu, focuses on channel hot-electron injection in MOSFETs [58]. This model was later challenged by the Energy-Driven Model (EDM) introduced in 2005, which emphasized the role of available energy over peak lateral electric field in predicting hot carrier effects in MOS devices. Furthermore, recent research has concentrated on electron–electron scattering-induced channel hot-electron injection in nanoscale n-MOSFETs with high-κ/metal gate stacks, highlighting the significance of trapping mechanisms in high-κ dielectric devices. Additionally, investigations on partially depleted SOI NMOSFETs revealed the impact of hot-electron injection on the back-gate threshold voltage and interface trap density, influencing the device’s direct-current characteristics and radiation hardness performance [59].

In the Lucky-Electron Hot Carrier Injection Model, it is proposed that an electron is emitted into the oxide by first gaining enough energy from the electric field in the channel to surmount the insulator/semiconductor barrier. Once the required energy to surmount the barrier has been obtained, the electrons are redirected towards the insulator/semiconductor interface by some form of phonon scattering. When these conditions are met, the carrier travelling towards the interface will then have an additional probability that it will not suffer any additional collision through which energy could be lost.

The model implemented into ATLAS is a modified version of the model proposed by Tam [58] and is activated by the parameters of HEI and HHI, for electron and hole injection, respectively, on the MODELS statement. The gate electrode–insulator interface is subdivided into several discrete segments which are defined by the mesh. For each segment, the lucky electron model is used to calculate the injected current into that segment. The total gate current is then the sum of all the discrete values.

If we consider a discrete point on the gate’s electrode–insulator boundary, we can write a mathematical formula for the current injected from the semiconductor. The formula calculates the injected gate current contribution from every node point within the semiconductor according to the injection current formula, stated as 2-dimensional integral of probability of hot electrons and holes, convolved with electron and current densities:

(12) $$I_{inj}=\iint P_{n}(x,y)\left|\;\vec{J_{n}}(x,y)\right|dxdy+\iint P_{P}(x,y)\left|\vec{{\;J}_{P}}(x,y)\right|dxdy$$

#### 3.2.3. Direct Quantum Tunneling Model

For deep submicron devices, the thickness of the insulating layers can be very small. For example, gate oxide thicknesses in MOS devices can be as low as several nanometers. In this case, the main assumptions of the Fowler–Nordheim approximation [60] are generally invalid and we need a more accurate expression for tunneling current. ATLAS used is based on a formula, which was introduced by Price and Radcliffe [61] and developed by later authors. It formulates the Schrödinger equation in the effective mass approximation and solves it to calculate the transmission probability, *T(E)*, of an electron or hole through the potential barrier formed by the oxide layer. The incident (perpendicular) energy of the charge carrier, *E*, is a parameter. It is assumed that the tunneling process is elastic. After considering carrier statistics and integrating over lateral energy, the formula

(13) $$J=\frac{qkT}{2\pi^{2}h^{3}}\sqrt{m_{y}m_{z}}\int T\left(E\right)ln\left\{\frac{1+e^{{(E}_{Fr}-E)/kT}}{1+e^{{(E}_{Fl}-E)/kT}}\right\}dE$$

is obtained, which gives the current density *J* (A/m^2^) though the barrier. The effective masses *m_y_* and *m_z_* are the effective masses in the lateral direction in the semiconductor. For example, for a direct bandgap material, where the Γ valley is isotropic, both *m_y_* and *m_z_* are the same as the density of states’ effective mass. The logarithmic term includes the carrier statistics and $E_{Fl}$ and $E_{Fr}\;$ are the quasi-Fermi levels on either side of the barrier. The range of integration is determined according to the band edge shape at any given contact bias [17].

#### 3.2.4. Employing the Computational Models in ATLAS

We model our gκ-FinFET using SILVACO ATLAS Deckbuild software tool. The family of such tools were used in vast amounts of research to design and simulate the MOSFET devices. ATLAS is actually a text-based language and takes an input file to be run to simulate the TFT devices. After building mesh and device geometry definitions, basic procedure for selecting mathematical models is adding the double line statement starting with keywords “**MODELS**” and “**INTERFACE**” to the ATLAS file, given in statement (14):

**MODELS** QTUNN.EL QTUNN.HO HEI HHI SCHRODINGER NEGF_MS SP.FAST SP.GEOM = 2DYZ **INTERFACE** TUNNEL (14)

By adding these within ATLAS file, researchers can employ direct quantum tunneling model (QTUNN.EL, QTUNN.HO) for both holes and electrons, hot-electron/hot-hole injection (HEI, HHI) model, non-equilibrium green function (NEGF_MS) model, and Schrodinger model [57] (SCHRODINGER), together with interface trap effect considerations simultaneously, to model complete current densities required for drain and gate leakage on any transistor with defined geometry, also defined in the ATLAS input (*.in) file. SP.FAST activates a fast product–space approach in a 2D Schrödinger solver. SP.GEOM = 2DYZ sets a dimensionality and direction of a Schrödinger solver. Value 2DYZ is default for mesh structure in ATLAS 3D.

### 3.3. Choice of Performance Metrics

Our performance metrics were selected, like in the paper by Nagy [31], for benchmarking of FinFETs, with DIBL added as the most researched short-channel effect, as follows:

i. I_G_, on-state gate leakage current, in Amperes, leaks from gate metal through dielectric into the channel, when V_GS_ = 1 V. In our case, we favor to minimize.
ii. I_ON_, on-state drain current, in Amperes, when V_DS_ = V_DD_ (= 1.25 V in our case) and V_GS_ = V_DD_. We favor to maximize.
iii. I_OFF_, off-state drain current, in Amperes, when V_DS_ = V_DD_ and V_G_ = −1.5 V. We favor to minimize.
iv. I_ON_/I_OFF_ ratio, unitless, accepted and powerful measure of TFT design quality. We favor to maximize.
v. V_TH_, threshold voltage, in Volts, the minimum V_GS_ voltage that drain current I_D_ slightly exceeds a limit current (1 × 10^−7^ A in our case) significant for the design. We favor to minimize.
vi. SS, Subthreshold Slope, in mV/decade, change in the gate voltage required a decrease in the drain current I_D_ by one decade, SS = ∆V_GS_/∆log (I_D_). We favor to minimize.
vii. DIBL, Drain-Induced Barrier Lowering, in mV/V, represents the drain voltage V_DS_ influence on the threshold voltage V_TH_, defined as DIBL = |∆V_TH_|/|∆V_DS_|. We favor to minimize.

as these are the primary FoMs for evaluation of thin film transistors’ performance, as also restated by Nowbahari [62] in his comprehensive review on junctionless transistors.

## 4. Results

We herein exhibit the performance of simulations carried out in ATLAS with the model given in Figure 1, of gκ-FinFET with gate oxide combinations tabulated in Table 4, in Figure 5, Figure 6, Figure 7, Figure 8, Figure 9, Figure 10, Figure 11, Figure 12 and Figure 13 and Table 5, Table 6, Table 7, Table 8, Table 9, Table 10, Table 11 and Table 12.

### 4.1. Drain Current Performance

**Figure 5:**
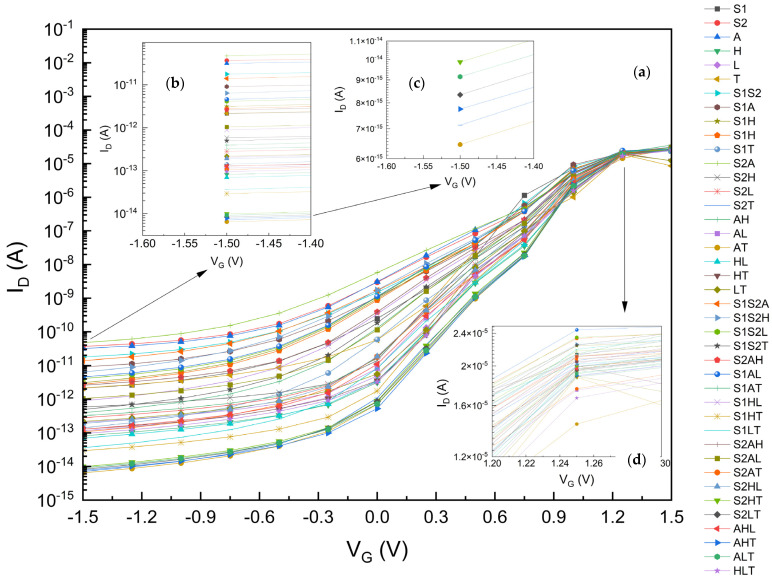
(**a**) Drain Current I_D_ for gκ-FinFETs with single, two-stage, and three-stage κ-graded gate oxides, (**b**) I_OFF_ zoomed for V_G_ between −1.6 and −1.4 V, (**c**) I_OFF_ further zoomed for V_G_ between −1.6 and −1.4 V, best six gκ-FinFETs, (**d**) I_ON_ zoomed for V_G_ between 1.2 and 1.3 V. See Table 7, Table 8 and Table 12 for summarized results of this figure.

First, our drain current modeling is verified by the results given in papers with FinFET fabrication examples [12,13,31,63]. Figure 5 shows the drain current I_D_ for all of single, two-stage and three-stage graded gate oxides for the gκ-FinFET device we examined, depicting the single and compounded performances of the SiO_2_, Si_3_N_4_, Al_2_O_3_, HfO_2_, La_2_O_3_, and TiO_2_ gate dielectrics. **S1AL** (SiO_2_: A_2_O_3_: La_2_O_3_) has the highest I_ON_ with 20.8 µA at (V_G_ = 1.25 V) performance. **AT** (Al_2_O_3_: TiO_2_ combination) has the lowest I_OFF_ current of 6.45 × 10^−15^ A. The I_OFF_ current significantly changed with the changing dielectric combination; it varied between 4.73 × 10^−11^ A and 6.45 × 10^−15^ A, more than four orders of magnitude, just because of modifying the gate oxide layer.

If a single layer was used, this range would be in between 2.14 × 10^−12^ A (for SiO_2_) and 8.18 × 10^−14^ A (for HfO_2_). The I_ON_ current would not be varying a great deal with changing gate oxides. However, gκ-FinFET **S2T** (Si_3_N_4_: TiO_2_ gate oxide) has the highest I_ON_ current of 2.08 × 10^−5^ A, better than any other single gate oxides including FinFET **H**. For I_ON_/I_OFF_, **S2T** also performed the best at 2.4 × 10^9^, one order higher than that of FinFET **H**.

As depicted in Figure 9, the best I_OFF_ performance gate oxides are **AT**, **S2T**, **AHT**, **S2LT**, and **ALT**, and from Figure 10, the best I_ON_ performance gate oxides are **S1AL**, **S1S2A**, **S1L**, **S1S2H**, **S1AH**, and **S1H**. We can observe that no single-material gate oxide has performed better than the two-stage or three-stage gate oxides in the drain current performances.

### 4.2. Leakage Current Performance

First, we observed that our gate leakage current model is verified as Rudenko [64], Garduno [32], Khan [65], and Golosov [66] have similar trends for I_G_: starting from a negative V_G_, I_G_ first decreases significantly around 6–14 orders of magnitude, depending on the gate oxide, takes a minimum at some V_G_ value, and then it increases steeply again.

**Figure 6:**
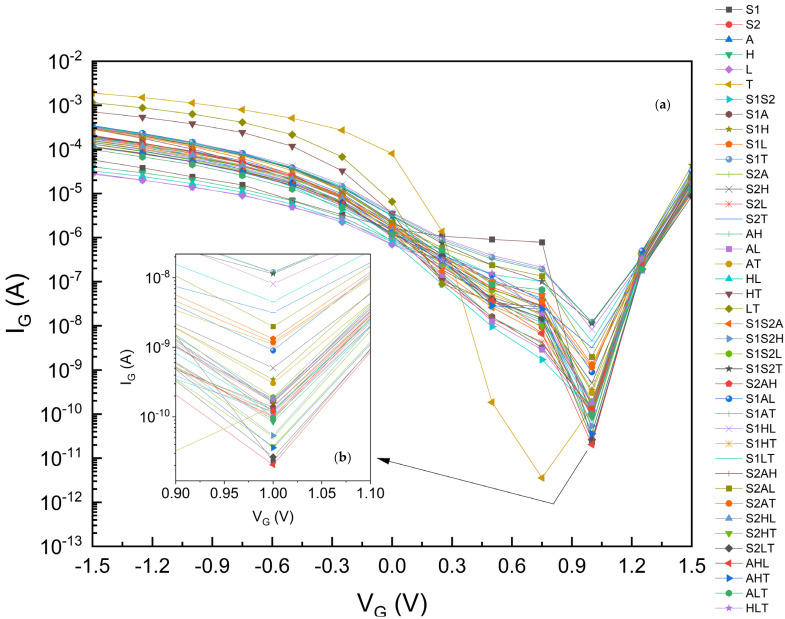
(**a**) Leakage Current I_G_ for gκ-FinFETs with single, two-stage, and three-stage κ-graded gate oxides, (**b**) I_G_ zoomed for V_G_ between 0.90 and 1.1 V. See Table 9 and Table 12 for summarized results of this figure.

Figure 6 shows the I_G_ leakage current characteristics [57] for the traditional single-material gate dielectrics together with the two-stage and three-stage κ-graded dielectrics, with the lowest gate leakage current of 2.04 × 10^−11^ A (20.4 pA) at V_G_ = 1.0 V for our specific FinFET under study. The leakage current curves generally show a similar trend and all tend to make local minimums at V_G_ = 1 V, with the exception of that of TiO_2_ which has a local minimum around V_G_ = 0.75 V and a leakage current of 4.0 × 10^−12^ A (4 fA). Despite this low leakage current, TiO_2_ does not behave well, especially regarding its DIBL, I_ON_, I_OFF,_ and I_ON_/I_OFF_ performance; thus, the sole usage of TiO_2_ as a gate dielectric cannot be advised.

### 4.3. DIBL, SS, I_ON_, I_OFF_, I_ON_/I_OFF,_ and V_TH_ Performance

**Figure 7:**
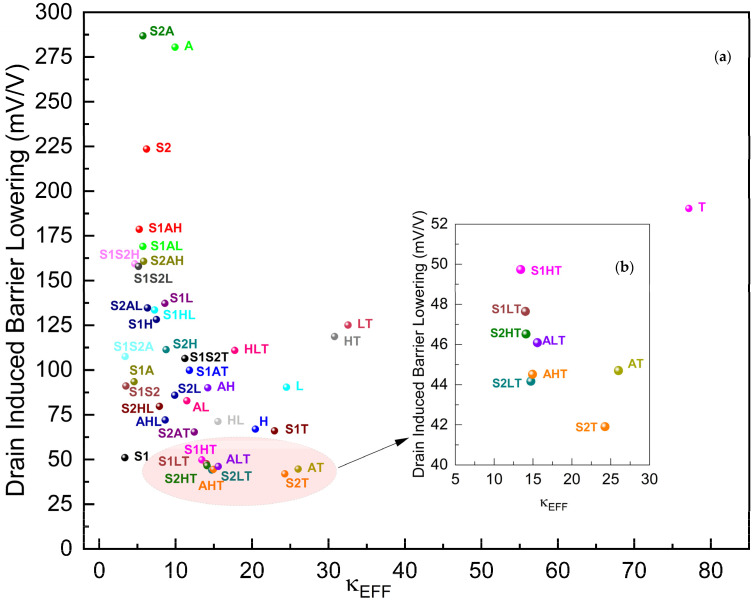
(**a**) DIBL of gκ-FinFETs with single, two-stage, and three-stage κ-graded gate oxides, (**b**) DIBL zoomed around $\kappa_{EFF}$ = 5~30. See Table 5 for concise results.

Figure 7 presents the Drain-Induced Barrier Lowering (DIBL) of FinFETs against their effective dielectric constants of gate oxides within. As DIBL is the short-channel effect where the drain voltage can influence the threshold voltage of the transistor, a lower DIBL value does generally better because it means the device has better control over the threshold voltage and is less susceptible to variations due to changes in the drain voltage.

The DIBL plot suggests that as the effective dielectric constant increases, the DIBL effect decreases steeply and significantly from $\kappa_{EFF}\;$≈ 3.35 until $\kappa_{EFF}\;$≈ 35, and then increases back until $\kappa_{EFF}\;$≈ 77, point **T** (designates FinFET with TiO_2_ as gate oxide). The DIBL performance of **S2T** with 41.9 mV/V is 37.4% lower than that of **H**. **S2T**, **S2LT**, **AHT**, **AT**, and **S2HT**, which are the five best-performing gκ-FinFETs in DIBL performance.

**Table 5:** DIBL of best 5 gκ-FinFETs versus the nearest performing single-layer configuration (**S1**).

	S2T	S2LT	AHT	AT	ALT	S1
DIBL (mV/V)	41.91	44.17	44.52	44.7	46.09	51.04
$\kappa_{EFF}$	24.26	14.71	14.39	24.87	15.02	3.35

**Figure 8:**
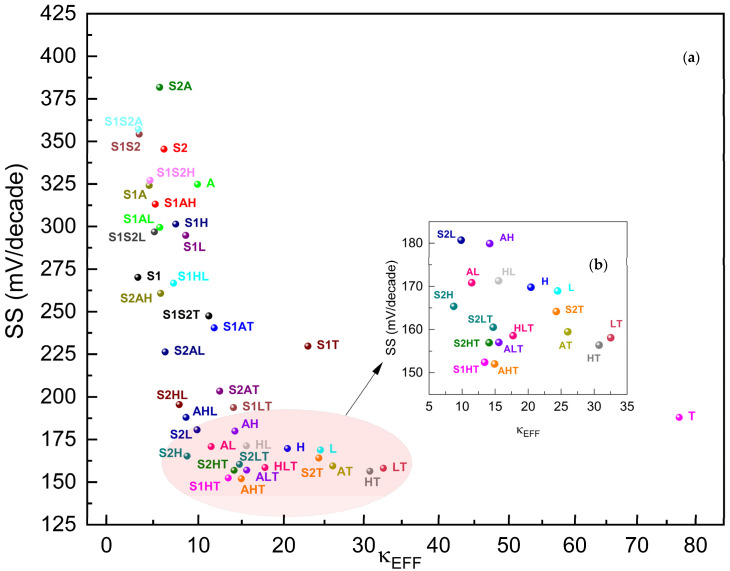
(**a**) SS of gκ-FinFETs with single, two-stage, and three-stage κ-graded gate oxides, (**b**) SS zoomed around $\kappa_{EFF}$ = 5~35. See Table 6 for concise results.

Figure 8 presents the Subthreshold Slope (SS) of gκ-FinFETs against their effective dielectric constants of gate oxides within. A lower SS means less change in the gate voltage is required to increase the drain current by a factor of ten. This is generally desirable as it indicates that the transistor can switch states more quickly and with less power consumption. Essentially, a lower subthreshold slope results in more efficient transistors that can operate effectively at lower voltages, which is especially beneficial in low-power and high-speed applications.

The SS plot suggests that as the effective dielectric constant increases, the SS effect decreases steeply and significantly from $\kappa_{EFF}\;$≈ 3.35 until $\kappa_{EFF}\;$≈ 25, just like DIBL’s regime, then increases almost linearly back until $\kappa_{EFF}\;$≈ 77, point **T** (designates gκ-FinFET with TiO_2_ as the gate oxide). **AHT**, **S1HT**, **HT**, **S2HT**, **ALT**, **HLT**, and **HT** are the best-performing FinFETs in SS performance. The SS performance of **AHT** with 152.0 mV/dec is 10.5% lower than that of **H**.

**Table 6:** Five best-performing gκ-FinFETs with lowest SS versus nearest-performing single-dielectric FinFET **L**.

	AHT	S1HT	HT	S2HT	ALT	L
SS (mV/dec)	152.0	152.41	156.39	156.93	158.57	164.17
$\kappa_{EFF}$	14.39	13.42	30.76	14.10	15.02	24.48

**Figure 9:**
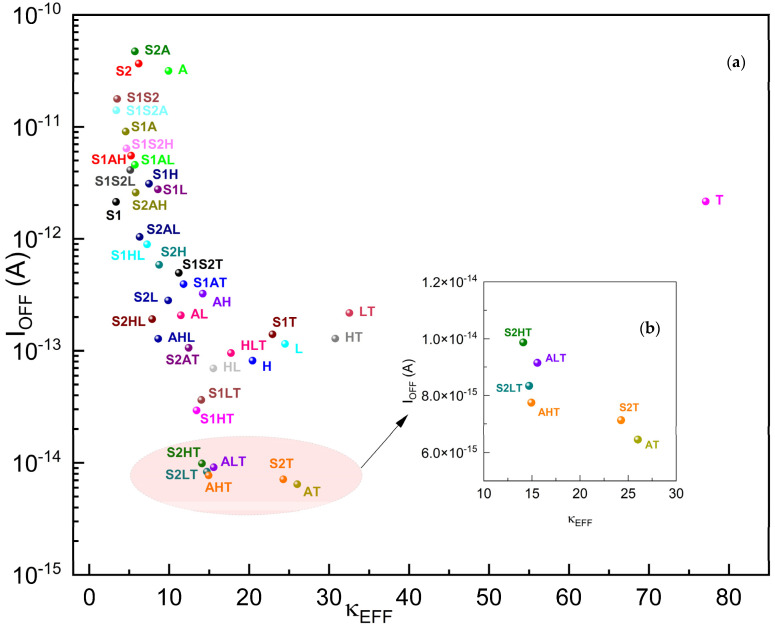
(**a**) I_OFF_ of gκ-FinFETs with single, two-stage, and three-stage κ-graded gate oxides, (**b**) I_OFF_ zoomed around $\kappa_{EFF}$ = 10~30. See Table 7 for concise results.

Figure 9 plots the I_OFF_ of gκ-FinFETs against the effective dielectric constant of gate oxides within. One of the primary advantages of a lower I_OFF_ is the decrease in power consumption, especially important in battery-powered devices like smartphones and laptops. When transistors leak less current in their off state, the overall power efficiency of the device improves, leading to a longer battery life and less heat generation. Also, with lower I_OFF_ values, it is possible to pack more transistors into a given area without significant overheating or power drain issues. This is critical for the ongoing trend of miniaturization in semiconductor technology.

The I_OFF_ plot suggests that as the effective dielectric constant increases, the I_OFF_ effect decreases steeply and significantly from $\kappa_{EFF}\;$≈ 3.35 until $\kappa_{EFF}\;$≈ 26 (that of **AT**), and then increases again until $\kappa_{EFF}\;$≈ 77. **AT**, **S2T**, **AHT**, **S2LT**, **ALT**, and **S2HT** are the best-performing gκ-FinFETs in I_OFF_ performance. The I_OFF_ performance of **AT** with 6.45 × 10^−15^ A is 92% lower than that of **H**.

**Table 7:** Five best-performing gκ-FinFETs with lowest I_OFF_ versus nearest-performing single dielectric FinFET **H**.

	AT	S2T	AHT	S2LT	ALT	H
I_OFF_ (A)	6.45 × 10^−15^	7.13 × 10^−15^	7.75 × 10^−15^	8.34 × 10^−15^	9.15 × 10^−15^	8.18 × 10^−14^
$\kappa_{EFF}$	24.87	24.26	14.39	14.71	15.02	20.43

**Figure 10:**
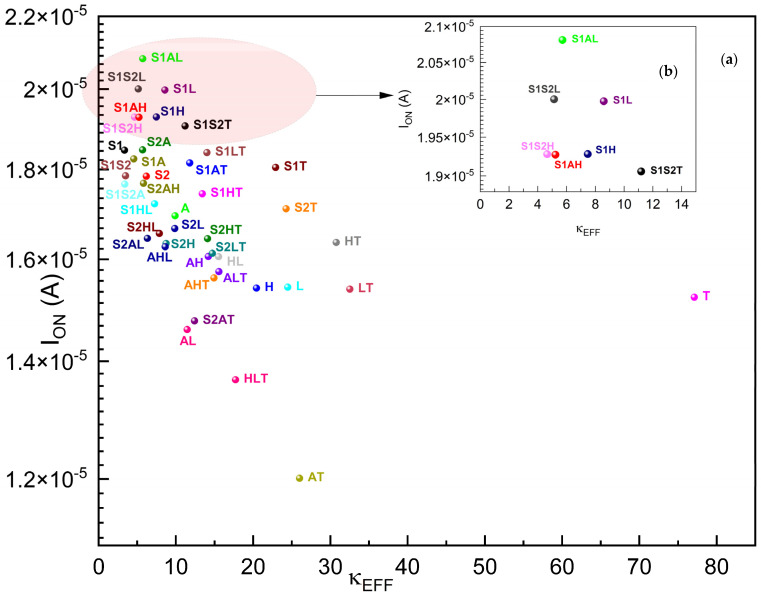
(**a**) I_ON_ of gκ-FinFETs with single, two-stage, and three-stage κ-graded gate oxides, (**b**) I_ON_ zoomed around $\kappa_{EFF}$ = 0~14. See Table 8 for concise results.

Figure 10 plots the I_ON_ of gκ-FinFETs against their effective dielectric constants of gate oxides within. A higher I_ON_ implies that the transistor can deliver more current rapidly, which generally translates to faster switching speeds. With a higher I_ON_, a transistor can drive larger currents through a circuit, which is essential for applications. The I_ON_ plot suggests that as the effective dielectric constant increases, the I_ON_ effect decreases steeply and significantly from $\kappa_{EFF}\;$≈ 3.35 until $\kappa_{EFF}\;$≈ 26 (that of **AT**), and then increases again until $\kappa_{EFF}\;$≈ 77, point **T**. **S1AL, S1S2L, S1L, S1S2H, S1AH,** and **S1AH** are the best-performing gκ-FinFETs in I_ON_ performance. The I_ON_ performance of **S1AL** is 2.4 × 10^8^, which is 35% higher than that of **H**.

**Table 8:** Five best-performing gκ-FinFETs with highest I_ON_ versus nearest-performing single dielectric FinFET **S1**.

	S1AL	S1S2L	S1L	S1S2H	S1AH	S1
I_ON_ (A)	2.081 × 10^−5^	2.000 × 10^−5^	1.998 × 10^−5^	1.928 × 10^−5^	1.927 × 10^−5^	1.846 × 10^−5^
$\kappa_{EFF}$	5.34	5.13	8.59	4.66	4.86	3.35

**Figure 11:**
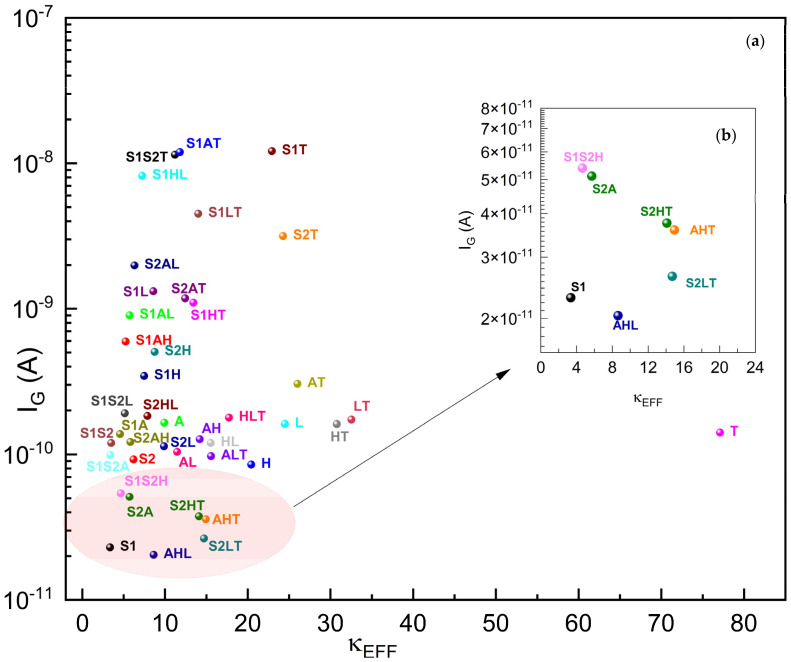
(**a**) I_G_ of gκ-FinFETs with single, two-stage, and three-stage κ-graded gate oxides, (**b**) I_G_ zoomed around $\kappa_{EFF}$ = 0~24. See Table 9 for concise results.

Figure 11 plots the I_G_ of gκ-FinFETs against their effective dielectric constants of gate oxides within. The I_G_ plot suggests that as the effective dielectric constant increases, the I_ON_ effect decreases steeply and significantly from $\kappa_{EFF}\;$≈ 3.35 until $\kappa_{EFF}\;$≈ 22 (that of **S1T**), and then increases again until $\kappa_{EFF}\;$≈ 77.

A lower I_G_ means the device has a better performance and less heating. A lower leakage current is preferable, especially for memory devices such as EEPROMs where a high I_G_ can contribute to charge loss and memory degradation over time [67,68,69]. With this fact in mind, **AHL**, **S1**, **S2LT**, **AHT**, **S2HT**, and **S1S2H** appear to be the best performers with respect to I_G_. Despite **S1**, all others are FinFETs with three-stage gate oxides, meaning κ-grading works properly in all cases.

We observe that no single-material gate oxide has performed better than the two-stage or three-stage gate oxides in leakage current performances. We find that the use of κ-graded stacked gate oxide dielectrics has the potential to generate lower gate-to-channel leakage currents, as stacked gate oxide **AHL** achieved a 76% lower I_G_ than the FinFET with a single HfO_2_ dielectric.

The performance of κ-graded gate oxides in terms of I_G_ appears to be better than that of single-material dielectrics, suggesting that κ-grading in gate oxides may provide a significant advantage in reducing I_G._ Also, they do not tend to exhibit any deficiency in device reliability, within the scope of this study.

**Table 9:** Five best-performing gκ-FinFETs with lowest I_G_ versus nearest-performing single dielectric FinFET **H**.

	AHL	S1	S2LT	AHT	S2HT	H
I_G_ (A)	2.04 × 10^−11^	2.29 × 10^−11^	2.65 × 10^−11^	3.59 × 10^−11^	3.76 × 10^−11^	8.53 × 10^−11^
$\kappa_{EFF}$	8.14	3.35	14.71	14.39	14.10	20.43

**Figure 12:**
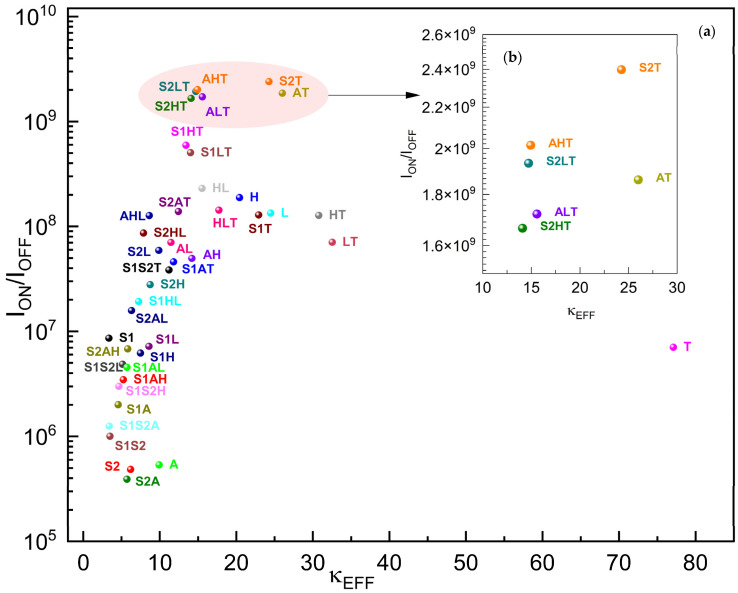
(**a**) I_ON_/I_OFF_ of gκ-FinFETs with single, two-stage, and three-stage κ-graded gate oxides, (**b**) I_ON_/I_OFF_ zoomed around $\kappa_{EFF}$ = 10~30. Observe Table 10 for concise results.

Figure 12 plots the I_ON_/I_OFF_ of the gκ-FinFETs against their effective dielectric constants of gate oxides within. A higher I_ON_/I_OFF_ is mostly desirable in any transistor application and it indicates a distinct and clear differentiation between the “on” and “off” states of the transistor. With a higher ratio, the transistor leaks significantly less current in the “off” state compared to the current it conducts in the on state. As transistors are miniaturized further, maintaining a high I_ON_/I_OFF_ ratio becomes increasingly important to ensure that the devices operate reliably without interference from leakage currents. It enables the continued scaling down of semiconductor devices following Moore’s Law, without performance degradation.

Our I_ON_/I_OFF_ plot suggests that as the effective dielectric constant increases, the I_OFF_ effect increases steeply and significantly from $\kappa_{EFF}\;$≈ 3.35 until $\kappa_{EFF}\;$≈ 24.26 (point **S2T**), and then decreases again until $\kappa_{EFF}\;$≈ 77. **S2T**, **AHT**, **S2LT**, **AT**, **ALT**, and **S2HT** are the best-performing gκ-FinFETs in I_ON_/I_OFF_ performance. The I_ON_/I_OFF_ performance of **S2T** is 2.4 × 10^9^, which is 11.73 times higher than that of FinFET **H**. We observe that no single-material gate oxide has performed better than the two-stage or three-stage gate oxides in I_ON_/I_OFF_ performance.

**Table 10:** Five best-performing gκ-FinFETs with lowest I_ON_/I_OFF_ versus nearest-performing single dielectric FinFET **H**.

	S2T	AHT	S2LT	AT	ALT	H
I_ON_/I_OFF_	2.40 × 10^9^	2.02 × 10^9^	1.93 × 10^9^	1.89 × 10^9^	1.72 × 10^9^	1.88 × 10^8^
$\kappa_{EFF}$	24.26	14.39	14.71	24.87	15.02	20.43

**Figure 13:**
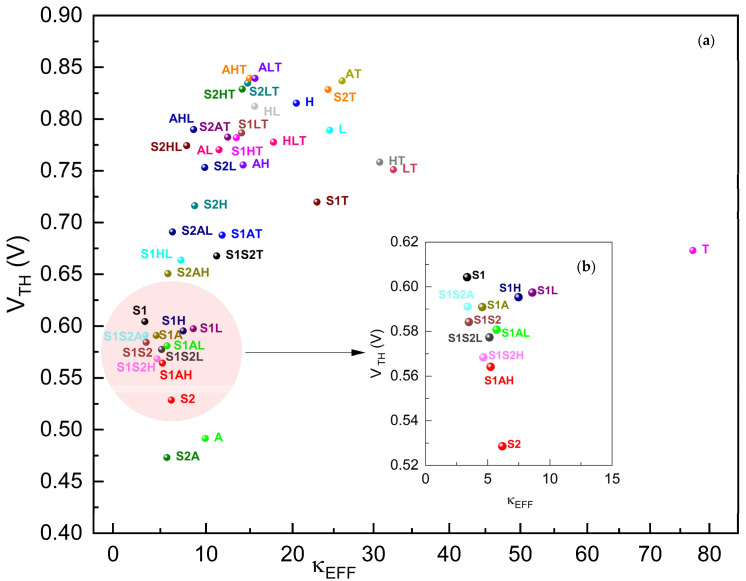
(**a**) V_TH_ of gκ-FinFETs with single, two-stage, and three-stage κ-graded gate oxides, (**b**) V_TH_ zoomed around $\kappa_{EFF}\;$ = 10~30. Observe Table 11 for concise results.

Figure 13 plots the V_TH_ of the gκ-FinFETs against their effective dielectric constants of gate oxides within. Devices with a lower V_TH_ can operate effectively at lower voltages. This is particularly advantageous in low-power applications such as mobile devices and wearable technology, where preserving battery life is crucial. A lower threshold voltage generally allows transistors to switch on and off more quickly. This can improve the overall speed of a processor and faster switching is beneficial for high-performance computing and digital circuits where rapid state changes are necessary.

The V_TH_ plot suggests that as the effective dielectric constant increases, the V_TH_ increases steeply and significantly from $\kappa_{EFF}\;$≈ 3.35 until $\kappa_{EFF}\;$≈ 26 (that of **AT**), and then decreases until $\kappa_{EFF}\;$≈ 77. **S2A**, **A**, **S2**, **S1AH**, **S1S2H**, **S1S2L,** and **S1AL** are the best-performing gκ-FinFETs in the V_TH_ performance. The V_TH_ performance of **S2A** with 0.4731 V is 3.76% lower than that of **A**, 10.5% lower than that of **S2,** and 42% lower than that of **H**. This shows how graded oxide is better than any other single dielectric, including **S2** and **A** individually, as shown in Table 6.

**Table 11:** Five best-performing gκ-FinFETs with lowest V_TH_ versus nearest-performing single dielectric FinFET **A**.

	S2A	A	S2	S1AH	S1S2H	S1S2L
V_TH_ (V)	0.4731	0.4916	0.5286	0.5641	0.5683	0.5773
$\kappa_{EFF}$	4.95	7.48	6.18	4.86	4.66	5.13

**Table 12:** FoM champions of gκ-FinFETs with two- and three-stage graded gate oxides compared with FinFET with single-layer HfO_2_ of t_ox_ 3 nm. Boldface indicates best value among all 41 gκ-FinFET configurations.

FoM	S2A	S2T	AT	S1AL	AHL	AHT	H
SS (mV/dec)	381.8	164.2	159.5	299.4	188.0	**152.0**	169.8
DIBL (mV/V)	286.9	**41.9**	44.7	169	72.1	44.52	66.9
I_ON_ (µA)	18.5	1.71	12	**20.8**	16.3	15.6	15.4
I_OFF_ (A)	4.73 × 10^−11^	7.13 × 10^−15^	**6.45 × 10^−15^**	4.58 × 10^−12^	1.28 × 10^−13^	7.75 × 10^−15^	8.18 × 10^−14^
I_ON_/I_OFF_ (×10^6^)	3.91	**240**	189	4.54	127	220	188
V_TH_ (V)	**0.4731**	0.8285	0.8369	0.5808	0.7899	0.8394	0.8153
I_G_ @ V_G_=1V (nA)	0.137	−3.16	−0.305	−0.9	**0.0204**	0.0359	0.085
$\kappa_{EFF}$	4.95	24.26	24.87	5.34	8.14	14.39	20.42

## 5. Discussion

As seen in Figure 10, the minimum I_OFF_ happens in gκ-FinFETs **AT**, **S2T**, **AHT**, **S2LT**, **ALT**, and **S2HT**. We observe that they have TiO_2_ in common. We may safely conclude that TiO_2_ matched perfectly with the metal side, better than others, and Al_2_O_3_ and Si_3_N_4_ matched (not so perfectly, but better than SiO_2_, HfO_2_, and La_2_O_3_) with the Si channel side when the FinFET was in depletion mode.

As seen in Figure 11, the maximum I_ON_ happens in gκ-FinFETs **S1AL**, **S1S2L**, **S1L**, **S1S2H**, **S1AH**, and **S1H**, and they all have SiO_2_ in common. We may also conclude that SiO_2_ matched perfectly with the Si channel side, better than the others and, La_2_O_3_ and HfO_2_ matched (not so perfectly, but better than Si_3_N_4_, Al_2_O_3_, and TiO_2_) with the metal side when the gκ-FinFET was in inversion mode.

All these observations and optimal values for all FoMs (Table 4) happen between $\kappa_{EFF}\;$ values of 4.95-24.87. Observing Figure 5 to 13, according to our findings, for the n+ Si family gκ-FinFETs, seeking dielectrics of $\kappa_{EFF}\;$ higher than 25 might not be so efficient as favorable FoM values all appear in the mentioned range of $\kappa_{EFF}$.

Therefore, it would be logical to infer, depending on the modes of the operation or the FoM we favor. In order to achieve this in a highly effective gate oxide layer, dielectric permittivity matching should be considered at both the neighboring Si channel side and neighboring gate metal side simultaneously.

This is the reason why we actually employed κ-graded stacked gate oxides, as their least dielectric permittivity side would match that of the Si channel side and the highest dielectric permittivity side of the same would match that of metal side, yielding lesser interface problems to widen the limits for a better gate oxide and transistor performance, while we restate the facts presented in the works of Giustino, Peng, and Wang [33,34,35,36]. We added below our insights which may lead to brief rules for designs in the future.

## 6. Analysis and Insights

Scanning throughout the 41 simulation results, we freely present our insights as follows:

- No obvious linear or quadratic relationship exists between composite gate oxide $\kappa_{EFF}\;$ and any of the FoMs examined; thus, a curve fitting was not possible.
- According to Table 7, the best I_OFF_ performances have a TiO_2_ laminate in common, as the last stage of the κ-graded structure. To minimize the I_OFF_, the dielectric permittivity of the gate metal and the neighboring gate oxide laminate should be kept as close as possible.
- According to Table 8, the best I_ON_ performances have a SiO_2_ laminate in common as the first stage of the κ-graded structure. To maximize the I_ON_, the dielectric permittivity of channel material and neighboring gate oxide laminate should be kept as close as possible.
- According to Table 11, the lowest values of V_TH_ appeared in the lowest values of $\kappa_{EFF}$.
- According to Table 12, the best DIBL performance appeared in the **S2T** (Si_3_N_4_: TiO_2_) gate oxide combination. To minimize the DIBL and maximize the I_ON_/I_OFF_, both the permittivity difference of the channel material and the neighboring gate oxide laminate, as well as the permittivity difference of the gate material and the neighboring gate oxide laminate should be kept small. In this case, the **S2T** gate oxide dielectric showed the perfect permittivity-matching behavior in between the neighboring Si and neighboring CrAu alloy.
- According to Table 12, at least one two-stage or three-stage κ-graded dielectric combination exists which will behave much better than all of the single-stage counterparts with respect to all our FoMs.

## 7. Fabrication Considerations

The deposition processes of the mentioned graded dielectric stack shown in Figure 1 should be achieved using the Atomic Layer Deposition (ALD) method so that thin films of the dielectric stack are obtained in an ALD reactor. ALD, a very slow process, will provide the deposition of thin film oxides with the thickness in order of a few angstroms, excellently uniform, accurate, and a pin-hole free [70,71]. Finally, the metal layer should be deposited by using magnetron sputtering or thermal evaporation onto the gate oxide layer [72].

## 8. Conclusions

We showed by simulations that it is possible that κ-graded stacked gate oxides could increase I_ON_ and reduce I_OFF_ and I_G_ currents, DIBL, SS, and V_TH_. A numerical analysis was conducted to show the viability of the usage of κ-graded dielectric structures against conventional single-layer high-κ dielectrics on a 14 nm FinFET geometry. The impact on the key electrical performance parameters is analyzed using SILVACO ATLAS as the device simulation tool. Within 41 different two- and three-stage κ-graded stacked gate oxide combinations, some FinFET structures with κ-graded gate oxides (gκ-FinFET) promise a lower gate leakage current I_G_ of up to 76%, lower drain-induced barrier lowering (DIBL) of up to 37.4%, a lower subthreshold slope (SS) of up to 10.5%, a lower drain-off current, I_OFF_, of up to 92%, a higher drain-on current, I_ON_, of up to 35%, a higher I_ON_/I_OFF_ ratio of up to 11.7 times, and a lower threshold voltage, V_TH_, of up to 42%, with respect to the FinFET of the same dimensions with a single-layer HfO_2_ gate dielectric. It became apparent that adverse interface effects will be minimized when smoother dielectric permittivity transitions are achieved by nanofabrication from the FinFET’s channel, up to its gate metal.

## Figures and Tables

**Table 1:** Simulated gκ-FinFET properties.

Property	Value	Note/Abbreviation
Channel (Fin) Length	14 nm	L_fin_
Gate thickness	1 nm	T_g_
Channel (Fin) Width	2 nm	W_fin_
Gate Length	14 nm	L_g_
Fin Width	2 nm	W_fin_
Fin Height	5 nm	H_fin_
Channel Concentration	5 × 10^19^ cm^−3^	N_d_
Gate work function	5 eV	*ϕ_w_*
Gate metal	CrAu alloy	-
FinFET Length	34 nm	L_FET_
FinFET Width	10 nm	W_FET_
Total Gate Oxide thickness	3 nm	t_ox_
Buried Oxide (BOX) Thickness	3 nm	t_BOX_
BOX material	HfO_2_	kept as is in all simulations
Bulk Si Thickness	10 nm	t_BULK_

**Table 4:** Effective dielectric constants κ_EFF_ of stacked nano-laminated gate oxides.

gκ-FinFET Reference Designator	Gate Oxide Material Thickness in nm							
	Total	SiO_2_	Si_3_N_4_	Al_2_O_3_	HfO_2_	La_2_O_3_	TiO_2_	$\kappa_{EFF}$
**S1**	3	3	-	-	-	-	-	3.35
**S2**	3	-	3	-	-	-	-	6.18
**A**	3	-	-	3	-	-	-	7.48
**H**	3	-	-	-	3	-	-	20.43
**L**	3	-	-	-	-	3	-	24.48
**T**	3	-	-	-	-	-	3	77.09
**S1S2**	3	1.5	1.5	-	-	-	-	3.48
**S1A**	3	1.5	-	1.5	-	-	-	3.86
**S1H**	3	1.5	-	-	1.5	-	-	7.48
**S1L**	3	1.5	-	-	-	1.5	-	8.59
**S1T**	3	1.5	-	-	-	-	1.5	22.92
**S2A**	3	-	1.5	1.5	-	-	-	4.95
**S2H**	3	-	1.5	-	1.5	-	-	8.73
**S2L**	3	-	1.5	-	-	1.5	-	9.86
**S2T**	3	-	1.5	-	-	-	1.5	24.26
**AH**	3	-	-	1.5	1.5	-	-	14.19
**AL**	3	-	-	1.5	-	1.5	-	10.43
**AT**	3	-	-	1.5	-	-	1.5	24.87
**HL**	3	-	-	-	1.5	1.5	-	15.53
**HT**	3	-	-	-	1.5	-	1.5	30.76
**LT**	3	-	-	-	-	1.5	1.5	32.53
**S1S2A**	3	1	1	1	-	-	-	3.07
**S1S2H**	3	1	1	-	1	-	-	4.66
**S1S2L**	3	1	1	-	-	1	-	5.13
**S1S2T**	3	1	1	-	-	-	1	11.19
**S1AH**	3	1	-	1	1	-	-	4.86
**S1AL**	3	1	-	1	-	1	-	5.34
**S1AT**	3	1	-	1	-	-	1	12.00
**S1HL**	3	1	-	-	1	1	-	7.24
**S1HT**	3	1	-	-	1	-	1	13.42
**S1LT**	3	1	-	-	-	1	1	14.02
**S2AH**	3	-	1	1	1	-	-	5.82
**S2AL**	3	-	1	1	-	1	-	6.31
**S2AT**	3	-	1	1	-	-	1	12.42
**S2HL**	3	-	1	-	1	1	-	7.86
**S2HT**	3	-	1	-	1	-	1	14.10
**S2LT**	3	-	1	-	-	1	1	14.71
**AHL**	3	-	-	1	1	1	-	8.14
**AHT**	3	-	-	1	1	-	1	14.39
**ALT**	3	-	-	1	-	1	1	15.02
**HLT**	3	-	-	-	1	1	1	17.73

## Data Availability

Data is available in the Supplementary Material.

## Supplementary Materials

The following supporting information can be downloaded at: https://www.mdpi.com/article/10.3390/mi15060726/s1.

## References

[1] Kol S., Oral A.Y. (2019). HF-based high-κ dielectrics: A review. Acta Phys. Pol. A.

[2] Baruah R.K., Paily R.P. (2013). Impact of high-k spacer on device performance of a junctionless transistor. J. Comput. Electron..

[3] Gieraltowska S., Wachnicki L., Witkowski B.S., Guziewicz E., Godlewski M. (2013). Thin films of high-k oxides and ZnO for transparent electronic devices. Chem. Vap. Depos..

[4] El N., Boukortt I., Hadri B., Patanè S. (2016). Effects of High-k Dielectric Materials on Electrical Characteristics of DG n-FinFETs. Int. J. Comput. Appl..

[5] Walker B., Pradhan A.K., Xiao B. (2015). Low temperature fabrication of high performance ZnO thin film transistors with high-k dielectrics. Solid. State Electron..

[6] Cheng Y.C., Chen H.B., Chang C.Y., Cheng C.H., Shih Y.J., Wu Y.C. A highly scalable poly-Si junctionless FETs featuring a novel multi-stacking hybrid P/N layer and vertical gate with very high Ion/Ioff for 3D stacked ICs. Proceedings of the Digest of Technical Papers-Symposium on VLSI Technology.

[7] Khatir A.M., Guen-Bouazza A., Bouazza B. (2013). 3D Simulation of Fin Geometry Influence on Corner Effect in Multifin Dual and Tri-Gate SOI-Finfets. Int. J. Nano Stud. Technol..

[8] Gomes T.C., Kumar D., Fugikawa-Santos L., Alves N., Kettle J. (2019). Optimization of the Anodization Processing for Aluminum Oxide Gate Dielectrics in ZnO Thin Film Transistors by Multivariate Analysis. ACS Comb. Sci..

[9] Hara A., Nishimura Y., Ohsawa H. Self-aligned metal double-gate junctionless p-channel low-temperature polycrystalline-germanium thin-film transistors with a thin germanium channel on a glass substrate. Proceedings of the AM-FPD 2016-23rd International Workshop on Active-Matrix Flatpanel Displays and Devices: TFT Technologies and FPD Materials.

[10] Nishimura Y., Nakashima T., Hara A. Self-aligned planar metal double-gate junctionless p-channel low-temperature poly-Ge TFTs with high-k gate dielectric on glass substrate. Proceedings of the International Display Workshops.

[11] Mustafa M., Bhat T.A., Beigh M.R. (2013). Threshold Voltage Sensitivity to Metal Gate Work-Function Based Performance Evaluation of Double-Gate n-FinFET Structures for LSTP Technology. World J. Nano Sci. Eng..

[12] Hisamoto D., Lee W.C., Kedzierski J., Takeuchi H., Asano K., Kuo C., Anderson E., King T.J., Bokor J., Hu C. (2000). FinFET-A Self-Aligned Double-Gate MOSFET Scalable to 20 nm. IEEE Trans. Electron. Devices.

[13] Bhattacharya D., Jha N.K. (2014). FinFETs: From Devices to Architectures. Adv. Electron..

[14] Sil M., Mallik A. (2021). On the logic performance of bulk junctionless FinFETs. Analog. Integr. Circuits Signal Process.

[15] Kuo Y. (1994). Thin Film Transistors with Graded SiN Gate Dielectrics. J. Electrochem. Soc..

[16] Dosev D., Iñíguez B., Marsal L.F., Pallares J., Ytterdal T. (2003). Device simulations of nanocrystalline silicon thin-film transistors. Solid. State Electron..

[17] Jankovic N. (2012). Numerical simulations of N-type CdSe poly-TFT electrical characteristics with trap density models of Atlas/Silvaco. Microelectron. Reliab..

[18] Kauerauf T., Govoreanu B., Degraeve R., Groeseneken G., Maes H. (2005). Scaling CMOS: Finding the gate stack with the lowest leakage current. in Solid-State Electron..

[19] Das R., Baishya S. (2019). Dual-material gate dual-stacked gate dielectrics gate-source overlap tri-gate germanium FinFET: Analysis and application. Indian J. Phys..

[20] Gangwani L., Hajela S. (2022). Analog Performance Analysis of a Novel 5nm Stacked Oxide Top Bottom Gated Junctionless FinFET. IOP Conf. Ser. Mater. Sci. Eng..

[21] Gardner M., Fulford J., Kwong D.-L. (2000). Method of Making Gate Dielectric for Sub-Half Micron MOS Transistors Including a Graded Dielectric Constant. U.S. Patent.

[22] Kang D., Genrik S., Kim C.-J., Park Y., Song I. (2011). Thin Film Transistor Having a Graded Metal Oxide Layer. U.S. Patent.

[23] Gealy D., Vishnavath B., Cancheepuram V.S., Rocklein M.N. (2012). US8110469 Graded Dielectric Structures.

[24] Veda S.N., Shaik H., Kumar K.N., Diwakar S.P., Kailash S., Chandrashekhar L.N., Pawar A.S. Simulation, Fabrication and Characterization of Zinc Oxide TFT. Proceedings of the 4th International Conference on Electronics, Communication and Aerospace Technology, ICECA 2020.

[25] Singh S., Chakrabarti P. (2012). Simulation, fabrication and characterization of sol-gel deposited ZnO based thin film transistors. Sci. Adv. Mater..

[26] Dwivedi R.D., Zhao Q. (2019). Simulation and Compact Modeling of Organic Thin Film Transistors (OTFTs) for Circuit Simulation. Int. J. Adv. Appl. Phys. Res..

[27] Bousari N.B., Anvarifard M.K., Haji-Nasiri S. (2019). Improving the electrical characteristics of nanoscale triple-gate junctionless FinFET using gate oxide engineering. AEU-Int. J. Electron. Commun..

[28] Vijaya P., Rohit L. (2022). Improvement of Ion, Electric Field and Transconductance of TriGate FinFET by 5 nm Technology. Silicon.

[29] Saha P., Dhar R.S., Nanda S., Kumar K., Alathbah M. (2023). The Optimization and Analysis of a Triple-Fin Heterostructure-on-Insulator Fin Field-Effect Transistor with a Stacked High-k Configuration and 10 nm Channel Length. Nanomaterials.

[30] Vimala P., Samuel T.S.A. (2020). TCAD Simulation Study of Single-, Double-, and Triple-Material Gate Engineered Trigate FinFETs. Semiconductors.

[31] Nagy D., Espineira G., Indalecio G., Garcia-Loureiro A.J., Kalna K., Seoane N. (2020). Benchmarking of FinFET, Nanosheet, and Nanowire FET Architectures for Future Technology Nodes. IEEE Access.

[32] Garduño S.I., Alvarado J., Cerdeira A., Estrada M., Kilchytska V., Flandre D. (2014). Gate leakage currents model for FinFETs implemented in Verilog-A for electronic circuits design. Int. J. Numer. Model. Electron. Netw. Devices Fields.

[33] Wang W., Li S. (2018). A transition of interface characteristics in LDPE/Al2O3 nanocomposites by permittivity simulation. IEEE Trans. Dielectr. Electr. Insul..

[34] Giustino F., Pasquarello A. (2005). Theory of atomic-scale dielectric permittivity at insulator interfaces. Phys. Rev. B Condens. Matter Mater. Phys..

[35] Peng S., Zeng Q., Yang X., Hu J., Qiu X., He J. (2016). Local Dielectric Property Detection of the Interface between Nanoparticle and Polymer in Nanocomposite Dielectrics. Sci. Rep..

[36] Wang W., Li S. Simulations of Effective Permittivity and Polarization Properties of Polyethylene Nanodielectrics. Proceedings of the IEEE International Conference on the Properties and Applications of Dielectric Materials (ICPADM).

[37] Fitio V., Vernygor O., Yaremchuk I. Analytical Approximations of the Noble Metals Dielectric Permittivity. Proceedings of the International Conference on Advanced Trends in Radioelectronics.

[38] Mei Z., Deng S., Li L., Wen X., Lu H., Li M. (2021). Dielectric function of sub-10 nanometer thick gold films. Appl. Phys. A Mater. Sci. Process.

[39] Roy B., Chakravorty D. (1993). High dielectric permittivity in glass-ceramic metal nanocomposites. Mater. Res. Soc..

[40] Phelps J.M., Taylor D.M. (1996). Determining the Relative Permittivity and Thickness of a Lossless Dielectric Overlayer on a Metal Film Using Optically Excited Surface Plasmon Polaritons. J. Phys. D Appl. Phys..

[41] Pavunny S.P., Misra P., Scott J.F., Katiyar R.S. (2013). Advanced high-k dielectric amorphous LaGdO3 based high density metal-insulator-metal capacitors with sub-nanometer capacitance equivalent thickness. Appl. Phys. Lett..

[42] Lucovsky G., Phillips J.C. (1998). Minimization of dangling bond defects in hydrogenated silicon nitride dielectrics for thin film transistors (TFTs). J. Non-Cryst. Solids.

[43] Lucovsky G., Phillips J.C. (1999). Why SiNx:H is the Preferred Gate Dielectric for Amorphous Si Thin Film Transistors (TFTS) and SiO2 is the Preferred Gate Dielectric for Polycrystalline Si TFTs. MRS Online Proc. Libr..

[44] Griep S., Luder E., Kallfab T. (1987). High Performance TFT of a-Si:H on a SiO2 Dielectric. J. Non-Cryst. Solids.

[45] Lee Y., Shin C. (2017). Impact of Equivalent Oxide Thickness on Threshold Voltage Variation Induced by Work-Function Variation in Multigate Devices. IEEE Trans. Electron. Devices.

[46] Li D.H., Park I.H., Cho S. Effects of Equivalent Oxide Thickness on Bandgap-Engineered SONOS Flash Memory. Proceedings of the IEEE Nanotechnology Materials and Devices Conference.

[47] Ni K., Saha A., Chakraborty W. Equivalent Oxide Thickness (EOT) Scaling With Hafnium Zirconium Oxide High-κ Dielectric Near Morphotropic Phase Boundary. Proceedings of the IEEE International Electron Devices Meeting (IEDM).

[48] Tienda-Luna I.M., Ruiz F.J.G., Donetti L., Godoy A., Gámiz F. (2008). Modeling the equivalent oxide thickness of Surrounding Gate SOI devices with high-κ insulators. Solid. State Electron..

[49] Chen C.H., Fang Y.K., Ting S.F., Hsieh W.T., Yang C.W., Hsu T.H., Yu M.C., Lee T.L., Chen S.C., Yu C.H. (2002). Downscaling Limit of Equivalent Oxide Thickness in Formation of Ultrathin Gate Dielectric by Thermal-Enhanced Remote Plasma Nitridation. IEEE Trans. Electron. Devices.

[50] Penn D.R. (1962). Wave Number Dependent Dielectric Function of Semiconductors. Electron. Transp. Mech. Insul. Film..

[51] Sharma A.C. (2006). Size-dependent energy band gap and dielectric constant within the generalized Penn model applied to a semiconductor nanocrystallite. J. Appl. Phys..

[52] Markel V.A. (2016). Introduction to the Maxwell Garnett approximation: Tutorial. J. Opt. Soc. Am. A.

[53] Azadmanjiri J., Berndt C.C., Wang J., Kapoor A., Srivastava V.K., Wen C. (2014). A review on hybrid nanolaminate materials synthesized by deposition techniques for energy storage applications. J. Mater. Chem. A.

[54] Tsu R., Babić D., Loriatti L. (1997). Simple model for the dielectric constant of nanoscale silicon particle. J. Appl. Phys..

[55] Niklasson G.A., Granqvist C.G., Hunderi O. (1981). Effective medium models for the optical properties of inhomogeneous materials. Appl. Opt..

[56] Petrovsky V., Jasinski P., Dogan F. (2012). Effective dielectric constant of two phase dielectric systems. J. Electroceram..

[57] Silvaco Inc. (1984). Atlas User Manual, Device Simulation Software, v5.34.0.R. www.silvaco.com.

[58] Tam S., Ko P.-K., Hu C. (1984). Lucky-Electron Model of Channel Hot-Electron Injection in MOSFET’s. IEEE Trans. Electron. Devices.

[59] Chen J.J., Chang T.C., Chen H.C., Zhou K.J., Kuo C.W., Wu W.C., Li H.C., Tai M.C., Tu Y.F., Tsai Y.L. (2020). Enhancing hot-carrier reliability of dual-gate low-Temperature polysilicon TFTs by increasing lightly doped drain length. IEEE Electron. Device Lett..

[60] Horiguchi S., Kobayashi T., Saito K. (1985). Interface-trap generation modeling of Fowler-Nordheim tunnel injection into ultrathin gate oxide. J. Appl. Phys..

[61] Price P.J., Rodcliffe J.M. (1959). Esaki Tunneling. IBM J. Res. Dev..

[62] Nowbahari A., Roy A., Marchetti L. (2020). Junctionless transistors: State-of-the-art. Electronics.

[63] Chen M.L., Sun X., Liu H., Wang H., Zhu Q., Wang S., Du H., Dong B., Zhang J., Sun Y. (2020). A FinFET with one atomic layer channel. Nat. Commun..

[64] Rudenko T., Kilchytska V., Collaert N., Jurczak M., Nazarov A., Flandre D. (2007). Reduction of gate-to-channel tunneling current in FinFET structures. Solid. State Electron..

[65] Khan A.A., Audhikary A., Al-Fattah F., Amin A., Nandi R. A Comparative Analytical Approach for Gate Leakage Current Optimization in Silicon MOSFET:A Step to More Reliable Electronic Device. Proceedings of the ICEEICT Conference.

[66] Golosov D.A., Vilya N., Zavadski S.M., Melnikov S.N., Avramchuk A.V., Grekhov M.M., Kargin N.I., Komissarov I.V. (2019). Influence of film thickness on the dielectric characteristics of hafnium oxide layers. Thin Solid Film.

[67] Wang B., Wang C.-H., Ma Y., Humes C.D.T. Study of Stress-Induced Leakage Current and Charge Loss of Nonvolatile Memory Cell 70A Tunnel Oxide Using Floating-Gate Integrator Technique. Proceedings of the IEEE International Integrated Relaibility Workshop.

[68] Schuler F., Degraeve R., Hendrickx P., Wellekens D. (2002). Physical charge transport models for anomalous leakage current in floating gate-based nonvolatile memory cells. IEEE Trans. Device Mater. Reliab..

[69] Lee D.U., Lee T.H., Kim E.K., Shin J.W., Cho W.J. (2009). Analysis of charge loss in nonvolatile memory with multi-layered SiC nanocrystals. Appl. Phys. Lett..

[70] Yu D., Yang Y.Q., Chen Z., Tao Y., Liu Y.F. (2016). Recent progress on thin-film encapsulation technologies for organic electronic devices. Opt. Commun..

[71] Lu Q., Yang Z., Meng X., Yue Y., Ahmad M.A., Zhang W., Zhang S., Zhang Y., Liu Z., Chen W. (2021). A Review on Encapsulation Technology from Organic Light Emitting Diodes to Organic and Perovskite Solar Cells. Adv. Funct. Mater..

[72] Li Y., Xiong Y., Yang H., Cao K., Chen R. (2020). Thin film encapsulation for the organic light-emitting diodes display via atomic layer deposition. J. Mater. Res..

